# Supplementary material for: Splice-Junction-Based Mapping of Alternative Isoforms in the Human Proteome
Source: Cell Rep. Author manuscript; Available in PMC 2020 Jan 15. (PMC6961840; doi:10.1016/j.celrep.2019.11.026)
Supplement: 2 [file NIHMS1546469-supplement-2.zip › DF1/PXD010154/Thyroid/Thyroid_12_TG_GLTPLEGTQDTFTNFQQVYLWK.pdf]

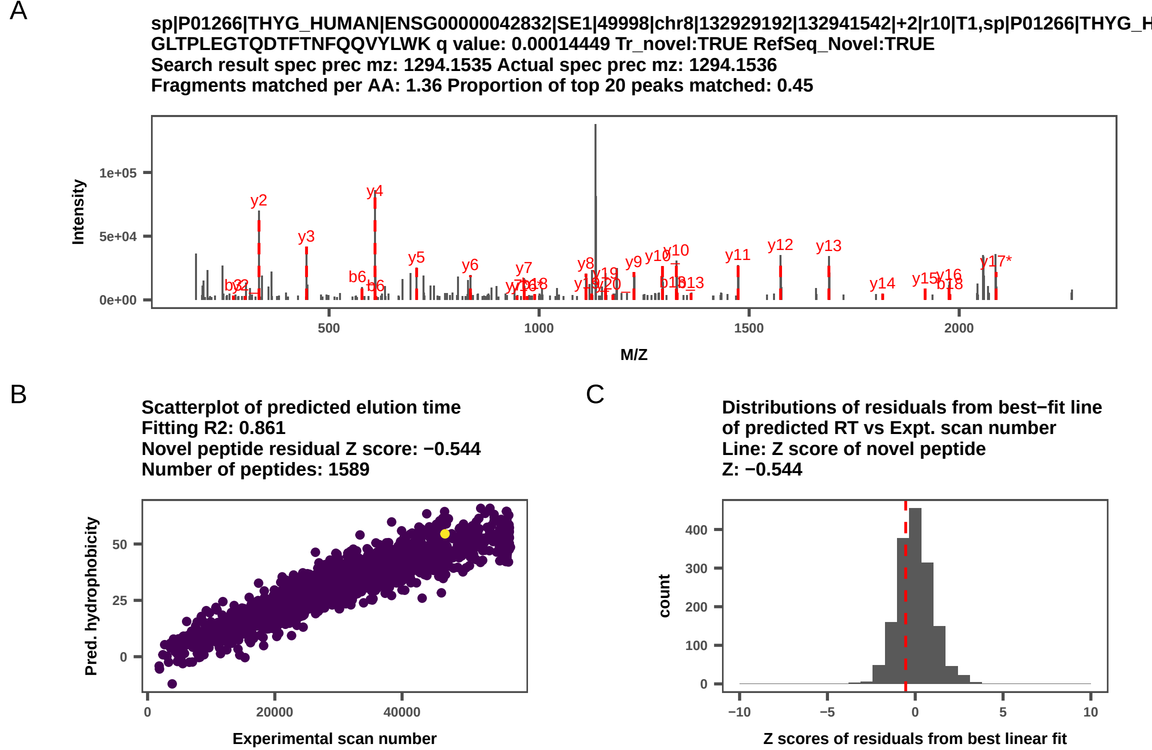
A
sp|P01266|THYG_HUMAN|ENSG00000042832|SE1|49998|chr8|132929192|132941542|+2|r10|T1,sp|P01266|THYG_H
GLTPLEGTQDTFTNFQQVYLWK q value: 0.00014449 Tr_novel:TRUE RefSeq_Novel:TRUE
Search result spec prec mz: 1294.1535 Actual spec prec mz: 1294.1536
Fragments matched per AA: 1.36 Proportion of top 20 peaks matched: 0.45
Intensity
1e+05
5e+04
0e+00
y2
y3
y4
y5
y6
y7
y8
y9
y11
y12
y13
y14
y17*
500
1000
1500
2000
M/Z
B
Scatterplot of predicted elution time
Fitting R2: 0.861
Novel peptide residual Z score: −0.544
Number of peptides: 1589
Pred. hydrophobicity
50
25
0
20000
40000
Experimental scan number
C
Distributions of residuals from best−fit line
of predicted RT vs Expt. scan number
Line: Z score of novel peptide
Z: −0.544
count
400
300
200
100
0
−10
−5
0
5
10
Z scores of residuals from best linear fit
